# The History of the Wolff–Parkinson–White Syndrome

**DOI:** 10.5041/RMMJ.10083

**Published:** 2012-07-31

**Authors:** Melvin M. Scheinman

**Affiliations:** University of California San Francisco, CA, USA

**Keywords:** Tachycardia, ventricular pre-excitation, Wolff–Parkinson–White syndrome

## Abstract

While Drs Wolff, Parkinson, and White fully described the syndrome in 1930, prior case reports had described the essentials. Over the ensuing century this syndrome has captivated the interest of anatomists, clinical cardiologists, and cardiac surgeons. Stanley Kent described lateral muscular connections over the atrioventricular (AV) groove which he felt were the normal AV connections. The normal AV connections were, however, clearly described by His and Tawara. True right-sided AV connections were initially described by Wood et al., while Öhnell first described left free wall pathways. David Scherf is thought to be the first to describe our current understanding of the pathogenesis of the WPW syndrome in terms of a re-entrant circuit involving both the AV node–His axis as well as the accessory pathway. This hypothesis was not universally accepted, and many theories were applied to explain the clinical findings. The basics of our understanding were established by the brilliant work of Pick, Langendorf, and Katz who by using careful deductive analysis of ECGs were able to define the basic pathophysiological processes. Subsequently, Wellens and Durrer applied invasive electrical stimulation to the heart in order to confirm the pathophysiological processes.

Sealy and his colleagues at Duke University Medical Center were the first to successfully surgically divide an accessory pathway and ushered in the modern era of therapy for these patients. Morady and Scheinman were the first to successfully ablate an accessory pathway (posteroseptal) using high-energy direct-current shocks. Subsequently Jackman, Kuck, Morady, and a number of groups proved the remarkable safety and efficiency of catheter ablation for pathways in all locations using radiofrequency energy. More recently, Gollob et al. first described the gene responsible for a familial form of WPW. The current ability to cure patients with WPW is due to the splendid contributions of individuals from diverse disciplines throughout the world.

While the eponym Wolff–Parkinson–White (WPW) syndrome is attributed to the landmark article published by the trio in 1930,^1^ other isolated case reports of the same entity were previously reported in the literature.^2^,^3^ Over the ensuing years this entity has captivated the interest of anatomists, clinical cardiologists, cardiac surgeons, clinical electrophysiologists, and more recently geneticists.

## ANATOMIC STUDIES

The story properly begins with the elucidation of the specialized conduction system of the heart. It was long appreciated that electrical connections bridged the atrial and ventricular chambers.^4^,^5^ Stanley Kent in 1893 described lateral atrioventricular (AV) connections and thought these constituted the normal specialized AV conduction system.^6^ This work proved controversial and was, in fact, rejected by such notables as Sir Thomas Lewis and Keith Flack. In a later study Dr. Kent described lateral connections with node-like structures which he felt constituted the normal AV conduction system.^7^

The work of His^8^ and Tawara^9^ clearly established the anatomy of the AV node and His–Purkinje system. Moreover, they proved that section of the His bundle resulted in complete AV block and described the His–Purkinje system.

It was clearly Wood et al.^10^ who first described the presence of a right-sided accessory pathway (AP) in a patient with an ECG pattern of pre-excitation and Öhnell who described left-sided APs in patients with pre-excitation.^11^ Other pioneer observations include those of Mahaim and Benatt who described connections between the AV node or His bundle to fascicles on ventricular myocardium.^12^

In an important study, Lev and Lerner^13^ presented a detailed investigation of 33 fetal and neonatal hearts and found no evidence for any lateral AV communications. They interpreted Kent’s finding of nodal tissue as being really atrial tissue: in neonates there is sparse collagen in the AV groove, and, depending on the angle of the sections, these may be misinterpreted as AV connections. It is, therefore, surprising that the eponym “Kent” bundle is still used to describe abnormal AV connections.

## CLINICAL AND INVASIVE ENDOCARDIAL STUDIES

Drs Wolff, Parkinson, and White are correctly credited with describing the entity that bears their names. Their article was published in the American Heart Journal in August 1930.^1^ They described 11 patients with short P-R and bundle branch block, who also suffered with paroxysmal supraventricular tachycardia (SVT) and/or atrial fibrillation (AF).

**Figure 1:**
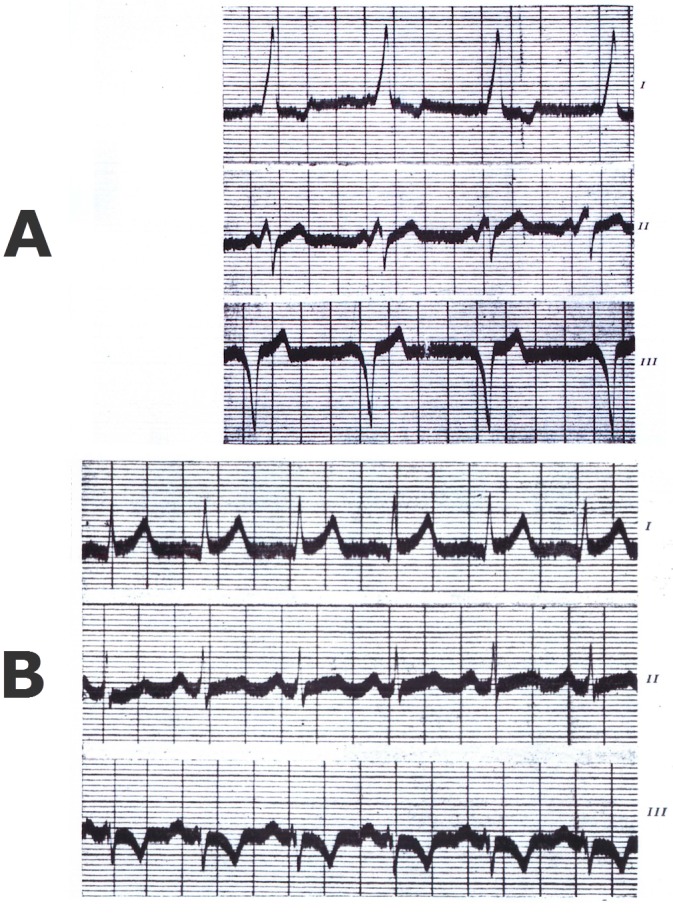
**Simultaneous recording of three-lead ECG obtained from the original article by Drs Wolff, Parkinson, and White.^1^ (Reproduced with permission from Elsevier.)** **A:** The ECG strips show clear-cut ventricular pre-excitation. **B:** The same patient is given intravenous atropine with normalization of the ECG. This finding was thought to explain the “neurogenic” bases for the Wolff–Parkinson–White syndrome. We now appreciate that facilitation of the atrioventricular nodal conduction with atropine may mask accessory pathway conduction. Alternatively, drugs that block atrioventricular nodal conduction may encourage accessory pathway conduction.

None of these patients had any evidence of structural cardiac disease, and the investigators confirmed (actually had reported previously) that administration of atropine would serve to normalize AV conduction with disappearance of the “bundle branch block” pattern (Figure 1). The authors felt that the entity was “neurogenic” in origin and was of little clinical consequence.

It was only later that Mines^14^ demonstrated that the concept of circus movement tachycardia was as a mechanism of tachycardia. According to T.N. James,^15^ it was Holzmann and Scherf^16^ in 1952 who were the first to describe pre-excitation as being due to antegrade conduction over an accessory AV connection.

The incredible contributions of Pick, Langendorf, and Katz deserve mention.^17^–^19^ They undertook detailed and painstaking analyses of literally thousands of strips from patients with the WPW syndrome and concluded that the arrhythmias were due to differences in conduction properties between the AV node and the AP, which allowed for initiation of SVT by premature beats. Remarkably they described concealed conduction into the pathway and the relationship between SVT and AF for these patients. Much of their pioneering observations were substantiated by intracardiac studies.

Drs Durrer and Wellens^20^,^21^ were the first to systematically use programmed electrical studies in numbers to clearly define the tachycardia mechanisms in patients with WPW. They showed that premature cardiac stimulation could induce orthodromic (SVT) (antegrade conduction over the AV node, retrograde conduction over the AP) as well as antidromic tachycardias (antegrade conduction over the AP, retrograde conduction over the node). These observations and others^22^,^23^ provided the framework for the use of intracardiac studies to define AP location and physiology.

## SURGICAL CONTRIBUTIONS

Prior to the current era of catheter ablation, patients with SVT intractable to drug therapy were treated with surgical dissection of the AV junction.^24^,^25^ This approach was largely used for management of the patient with atrial fibrillation refractory to drug therapy but would not be appropriate for those with APs since extirpation of the AV junction would not mitigate against rapid conduction over an AP. Durrer and Roos^26^ performed intraoperative mapping and cooling (in an important proof of concept experiment) to locate and transiently prevent conduction in a patient with a right-sided AP. Subsequently Burchell et al.^27^ used intraoperative mapping and abolished pre-excitation with a local injection of procainamide. A limited surgical incision over this area resulted in only transient loss of pre-excitation. Sealy et al.^28^ were the first to successfully ablate an AP in a human. The Duke team initially used an epicardial approach but subsequently showed that APs in all locations (both free wall and septal) could be successfully ablated using an endocardial technique.^29^ Only later was a cryo-epicardial technique used by Guiraudon et al.^30^

## CATHETER ABLATION

The technique of catheter ablation of the AV junction was introduced by Scheinman et al. in 1981.^31^ The technique involved use of high-energy direct-current shocks delivered to the region of the AV junction. This was followed by attempts to use catheter techniques for ablation of APs in various locations. In 1984 Fisher et al.^32^ used this technique for attempted ablation of left-sided APs via the coronary sinus. This technique was abandoned due to limited efficacy and risk of cardiac tamponade. In 1984 Morady and Scheinman^33^ reported a catheter technique for right posteroseptal APs associated with a 65% efficacy without cardiac tamponade as the shock was delivered just outside the os of the coronary sinus. In addition Warin et al. described successful catheter ablation of free wall APs using high-energy D/C shock.^34^

**Figure 2:**
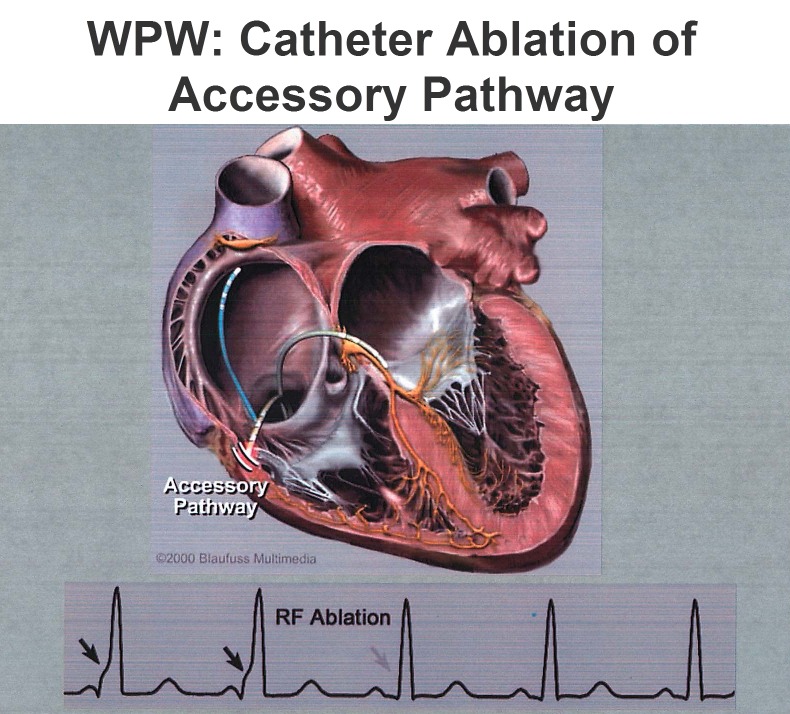
**Schema showing use of catheter technique for ablation of a right free wall accessory pathway.** The rhythm strip shows disappearance of the delta wave with application of radiofrequency energy. (Reproduced with permission from Blaufuss Multimedia.)

The subsequent introduction of radiofrequency energy for catheter ablation^35^ completely revolutionized our approach to the management of patients with WPW (Figure 2). Use of radiofrequency energy, as well as improved mapping and catheter design, has had a dramatic impact on patient management. The remarkable work particularly of Jackman et al. introduced techniques of both recording and ablation of AP potentials.^36^ The modern era of widespread use of radiofrequency ablation for patients with AP-mediated tachycardia was documented by the pioneering efforts of several groups.^36^–^38^ Moreover, the efficacy and safety of these procedures have been documented by registry and prospective studies.^39^,^40^

## FUTURE DIRECTIONS

Catheter ablative procedures have become the method of choice for care of patients with the WPW syndrome. While incremental improvements in catheter design or mapping systems will undoubtedly facilitate ablative procedures, the major advances appear to reside in the area of molecular genetics and biology. Mehdirad et al.^41^ described an autosomal dominant form of WPW associated with cardiomyopathy and progressive cardiac conduction system disease linked to chromosome 7q3. Subsequently Gollob et al.^42^ identified a missense mutation in the gene that encodes the gamma-2 regulatory subunit of AMP-activated protein kinase which was associated with the WPW syndrome in two families. These families were characterized as having cardiomyopathy, atrial fibrillation, multiple APs, and a poor prognosis. Protein kinase is involved in the phosphorylation of multiple metabolic pathways including energy substrate regulation. The genetic abnormality has been associated with cardiac glycogen storage disease.^43^

Further studies have defined the role of epicardial derived cells in the formation of the AV groove.^44^ Conceivably, defects in function of these primitive cells act as progenitor for residual muscle connections between atrium and ventricle. More recently studies involving activation of notch signaling provided fully penetrant APs as well as ventricular pre-excitation in the developing mouse heart.^45^ Alternatively, inhibition of notch signaling leads to a hypoplastic AV node with loss of slowly conducting cells.

## CONCLUSION

The current history of the WPW syndrome results in a happy situation where a curative procedure is available for the majority of our patients. This situation arose from the brilliant collaborative work of anatomists and clinicians who described the syndrome, as well as surgeons and cardiac electrophysiologists. Further advances in understanding the precise pathogenesis of this disorder belong to the molecular biologists.

## Abbreviations:

AF: atrial fibrillation;
AP: accessory pathway;
AV: atrioventricular;
SVT: supraventricular tachycardia;
WPW: Wolff-Parkinson-White.
